# Reporting at Least One Adverse Effect Post-COVID-19 Vaccination From Primary Health Care in Muscat

**DOI:** 10.7759/cureus.17055

**Published:** 2021-08-10

**Authors:** Thamra S Al Ghafri, Lamya Al Balushi, Zainab Al Balushi, Fatma Al Hinai, Said Al Hasani, Huda Anwar, Muna Al Lawati, Saud Al Harthi

**Affiliations:** 1 Primary Care, Oman Ministry of Health, Muscat, OMN; 2 Department of Disease Surveillance and Control, Oman Ministry of Health, Muscat, OMN; 3 Disease Surveillance and Control, Oman Ministry of Health, Muscat, OMN; 4 Planning and studies, Oman Ministry of Health, Muscat, OMN; 5 Planning and Studies, Oman Ministry of Health, Muscat, OMN; 6 Family Medicine, Ministry of Health, Muscat, OMN; 7 Quality and Patient Safety, Oman Ministry of Health, Muscat, OMN; 8 Medicine, Al Nahdha Hospital, Muscat, OMN

**Keywords:** covid-19, pfizer, astrazeneca, side effects, vaccines, primary care, vaccination, correlates

## Abstract

Introduction

Vaccinations against COVID-19 were licensed with limited testing assurances to the public triggering a widespread hesitancy around expected adverse reactions. Limited data was reported from Arabian Gulf countries on vaccine adverse effects.

Objectives

This study looked at the rate of reporting at least one side effect post-COVID-19 vaccination and its associated factors (sociodemographic characteristics, clinical condition, and type of vaccines). Additionally, questions about safety and willingness to recommend them were included.

Study design

Phone interviews on post-COVID-19 vaccination adverse effects were utilized to record responses related to reporting at least one side effect post vaccinations across the studied variables. Data collection continued for two months (from 1^st^ March to 30^th^ April 2021).

Methodology

Participants were adults (Omani citizens and non-citizens) who received AstraZeneca (AZ) or Pfizer (PF) vaccines from primary care facilities in Muscat and were randomly selected from the health information system. Responses were saved in a bespoke Google form/questionnaire. Chi-squared tests were utilized to determine potential factors associated with the dependent variable.

Results

A total of 753 participants completed the phone interviews. The mean age was 52 (3.5), males (54.1%), and 65.1% were Omanis. Hypertension (39.7%), diabetes (34.1%), and asthma (16.7%) were the commonest comorbidities. AZ and PF were administered to 78% and 22% of the participants. Of them, 49.8% reported at least one adverse effect post-COVID-19 vaccination.

The proportion of participants with at least one adverse effect was significantly more in individuals who were younger, females, with more than secondary education, and employed (p value < 0.001, 0.01, <0.001, and <0.001, respectively). There was no severe reaction (anaphylactic shock) to the vaccines, and most adverse effects were mild-moderate. The proportion of individuals who reported adverse effects were higher with AZ vs PF (53% vs 38.6, p = 0.001). The most common reported localized adverse effects were pain and tenderness (28.3% and 12.1%). Fever and body aches were the commonly reported systemic adverse effects (33.5% and 29.2%).

The safety of COVID-19 vaccines was well perceived, and most participants were willing to recommend them to others.

Conclusions

The current study confirms findings from existing literature on the mild to moderate adverse effects of AZ and PF vaccines. Despite the subjective nature of this study, it is reassuring that the studied COVID-19 vaccines can be administered safely. However, more longitudinal studies are needed to test their efficacy in disease prevention.

## Introduction

The December 2019 coronavirus (COVID-19) that was reported in one country continued to spread globally. Non-pharmaceutical intervention (NPI) of different scales was imposed globally [1]. After one year following the outbreak, there is no consensus on the best treatment for the severe forms of COVID-19 within the medical community. However, vaccinations were reported to be effective in reducing COVID-19 mortalities [2].

To contain the spread of the virus, NPIs, including traditional social distancing, quarantine, use of disinfectant substances, and wearing protective face masks, were widely practiced [3]. These measures have adverse consequences, both psychological and economical, and have resulted in substantial disagreement among the medical community and political decision-makers regarding their efficacy [3,4]. Along with the restrictions and anti-viral treatments, the production of vaccines has been accelerated. Questions about safety and potential adverse vaccine effects were raised due to accelerated vaccine development [5].

On the 11^th^ of August 2020, a Sputnik-5 vaccine was approved by the Ministry of Health of the Russian Federation. Accelerated vaccine production suggests that safety testing was performed in ≤ one year, a time frame significantly shorter than the accepted periods [6]. It is postulated that it may be difficult to see how mid- and long-term safety testing for the proposed vaccine (or any vaccine or drug) can be performed credibly in such a short time frame [5]. Vaccines work by stimulating the body's immune system to recognize and fight off the viruses and bacteria they target. After vaccination, if the body is later exposed to those germs, it is promptly ready to destroy them, preventing illness [7].

As of 18^th^ February 2021, at least seven different vaccines were introduced globally. Vulnerable populations in all countries were prioritized for vaccination. Vaccines were reported as an essential tool to fight COVID-19, and it is hugely encouraging to see so many vaccines proving successful and going into development [8]. A nationwide mass vaccination study suggested that the BNT162b2 mRNA vaccine was effective for a wide range of COVID-19-related outcomes [9]. However, risks for adverse effects post vaccinations differed across studies, and further longitudinal studies are warranted [10]. The most common reported adverse effects following vaccination with the Pfizer (PF) and Moderna's messenger RNA or mRNA vaccines are soreness at the injection site. Other adverse effects include fatigue, headache, muscle aches, chills, joint pain, and possibly some fever. Adverse effects were more frequent after the second dose in the vaccine trials. Adverse effects are similar after the PF and Moderna mRNA vaccines but could differ from other types of vaccines. These adverse effects are typical of the inflammation induced by vaccines and signify the body’s immune response to the vaccine [11,12].

In Oman, vaccinations, namely PF, started on the 27^th^ of December 2020 and Oxford AstraZeneca (AZ) on the 7^th^ of February 2020. Ministry of Health implemented a standardized electronic system "Tarassud" as a vaccination registry, and passive vaccine adverse event surveillance and reporting were done using the same system. However, no structured analysis has been done so far. During mass vaccination campaigns, adverse events are likely to generate concerns among the community, which may lead to hesitancy for vaccination. Notably, enhancing active surveillance of adverse events can provide scientific evidence to describe the reported adverse events that are mostly mild, self-limiting, and treated with pain relievers [13].

This study aimed at determining the rate of reporting at least one adverse effect post-vaccination. Additionally, it described the common adverse effects reported by individuals who received the COVID-19 vaccines. Also, correlates, defined as factors (socio-demographics, clinical, vaccine type, and number doses) associated with reporting adverse effects post-vaccination, were studied. Finally, perceptions on safety, effectiveness, and willingness to recommend COVID-19 vaccination to others were equally explored.

## Materials and methods

This was a cross-sectional analytical study specifically designed to gather information on post-COVID-19 vaccinations administered to Omani adults in primary care facilities in Muscat, Oman, from 1^st^ March to 30^th^ April 2021. Items of the survey were discussed among various public health and clinical experts considering the sociocultural aspect of Oman. The survey was piloted on 10 individuals who had taken the vaccine outside the sampled study population. internal consistency reliability measures were investigated through the use of factor analysis using SPSS v22 (IBM Corp., Armonk, NY). Cronbach’s alpha test [14] was 0.82 indicating good internal consistency of the scale in this study population. The items of the survey were adjusted and corrected after the piloting.

The survey included sociodemographic data, basic epidemiological and clinical data, history of COVID-19 exposure, vaccination (type) history, the incidence, and the severity (defined by requiring medical attention) of the respective adverse effects. Adverse effects were acknowledged as follows: (a) localized reactions (pain, swelling, tenderness, redness, itching, or other) or (b) systemic reactions (fever; skin rash; shortness of breath; tingling in the mouth, face, body/extremities; swelling in the face or mouth; generalized swelling; anaphylaxis/severe allergic reaction with low blood pressure, face swelling, and breathlessness; tiredness or fatigue; flu-like illness; or any other adverse effects). Additional two close-ended (yes or no) questions were included on perceptions on vaccine safety, and willingness to recommend them to others was included to assess vaccine acceptance.

Due to physical distancing and restrictions to prevent COVID-19, the participants were contacted via phone interviews by trained recruiters (described later) and responses were recorded utilizing a bespoke Google form/questionnaire.

Inclusion criteria

All participants (Omani and non-Omani) who received the first or second dose of the COVID-19 vaccine (PF or AZ) in primary healthcare were included. Administration of the vaccine had to be at least seven days before data collection of this survey. Participants were asked to give verbal consent prior to the phone interview.

Exclusion criteria

Participants from other than primary healthcare vaccine centers from other governorates were excluded.

Sample size and sampling technique

According to the electronic health information system, a total of 18,957 were vaccinated in primary health centers in the Muscat governorate until the 15^th^ of March 2021. Assuming that 2% of the subjects in the population have the factor of interest [15], 95% confidence limits, a response rate of 80%, and a precision of 20%, the calculated sample size was 753 participants. Participants were required to be recruited with 5% absolute precision and 95% confidence. The required sample size was selected randomly using randomly generated tables in SPSS v21.

Ethics

Ethical approval was obtained from the Regional Ethical Committee (NH/DGHS/P&S/2/2021).

Training and data collection

Existing healthcare workers (two administrators, three doctors, four public health workers) were recruited to conduct the telephone interviews after a one-day training on the study assessment tools. Phone interviews lasted seven to 15 minutes, and responses were recorded simultaneously. Calls were limited to five attempts. Wrong phone numbers and non-responding participants were replaced by new participants from the available randomized list. A total of 1000 vaccinated clients were called.

Analysis

Descriptive statistics were expressed as mean (SD) or percentages. The dependent variable of reporting at least one adverse effect was assessed across various sociodemographic characteristics, clinical status (existing comorbidities), type, and the number of doses of the administered COVID-19 vaccine. The chi-squared test was utilized to identify the significant factors associated with the dependent variable (p < 0.05). All analyses were done via SPSS v21.

## Results

Sociodemographic characteristics of participants

A total of 753 participants completed the phone interviews. The mean age was 62 (SD = 3.5), with more than half of the population ≥60 years of age (58.4%, n = 440). There were slightly more males (54.1%, n = 407) than females (45.9%, n = 346), 65.1% were Omani, 59.1% (n = 445) had more than secondary education, 65.8% (n = 496) were not employed, and most of them lived within a single family (78.6%, n = 592) (Table 1).

**Table 1:** Results of reporting at least one adverse effect post-COVID-19 vaccination across the sociodemographic characteristics of the study participants

Sample characteristics	Total sample, N = 753 (%)	Reporting at least one adverse effect post-COVID-19 vaccination		P value
		No, n = 378 (50.2%)	Yes, n = 375 (49.8%)	
Age (in years)				<0.001
< 60	313 (41.6)	105 (38.0)	208 (62.0)	
≥ 60	440 (58.4)	273 (62.0)	167 (38.0)	
Gender				0.010
Female	346 (45.9)	156 (45.1)	190 (54.9)	
Male	407 (54.1)	222 (54.5)	185 (45.5)	
Nationality				0.763
Non-Omani	263 (34.9)	134 (51.0)	129 (49.0)	
Omani	490 (65.1)	244 (49.8)	246 (50.2)	
Educational level				<0.001
≤ Secondary education	272 (40.9)	195 (63.3)	113 (36.7)	
> Secondary education	445 (59.1)	183 (41.1)	262 (58.9)	
Employment				<0.001
Unemployed	496 (65.8)	255 (56.8)	194 (43.2)	
Employed	257 (34.2)	107 (41.6)	150 (58.4)	
Living status				0.100
Living alone	52 (6.9)	27 (51.9)	25 (48.1)	
One family	592 (78.6)	289 (48.8)	303 (51.2)	
Extended family or group accommodation	108 (14.5)	62 (57.4)	46(45.6)	

The most common chronic conditions reported by the participants were hypertension (HTN) (39.7%), diabetes (DM) (34.1%), asthma (16.7%), and history of allergy (13.2%).

Administered vaccines and commonly reported adverse effects

Out of the 753 participants, 587 (78%) received AZ, and 166 (22%) received PF as the first and/or second dose. Almost half of them (49.8%) reported at least one side effect post-COVID-19 vaccination. In general, most reported adverse effects were mild to moderate and resolved within ≤ seven days.

Generally, only 20.8% of the participants received two doses of the vaccines, 35.5% (n = 59) of PF and 16.7% (n = 98) of AZ. Only 4.6% (n = 35) of the participants reported a positive history of confirmed COVID-19 before vaccination (two weeks to three months before vaccination); however, none of them required hospital admission.

No significant association was found between reporting at least one adverse effect post-COVID-19 vaccination and previous history positive polymerase chain reaction (PCR) for severe acute respiratory syndrome coronavirus 2 (SARS-CoV-2) (Table 2). The proportion of participants who reported adverse effects was significantly higher among participants who received AZ vs PF (53% vs 38.6% respectively, p = 0.001) (Table 2). Almost three-fourth of the participants received one dose of any of the vaccines. The proportion of participants who reported at least one adverse effect was significantly more among those who received two doses vs one dose of any vaccine (60.5% vs 47.0% respectively, p value = 0.004) (Table 2). However, more adverse effects were reported after the second dose of the PF, while AZ recipients reported them more frequently after the first dose.

**Table 2:** Results of reporting at least one adverse effect post-COVID-19 vaccination across type and dose of the administered vaccines

Sample characteristics	Total sample, N = 753 (%)	Reporting at least one side effect post-COVID-19 vaccination		P value
		No, n = 378 (50.2%)	Yes, n = 375 (49.8%)	
Type of vaccination				0.001
AstraZeneca	587 (78.0)	276 (47.0)	311 (53.0)	
Pfizer	166 (22.0)	102 (61.4)	64 (38.6)	
Number of doses				0.004
1^st^ dose	596 (79.2)	316 (53.0)	280 (47.0)	
2^nd^ dose	157 (20.8)	62 (39.5)	95 (60.5)	

Reported adverse reactions after COVID-19 vaccination

Overall, almost all participants (93.9%) reported adverse effects within 48 hours post-vaccination. The most common reported localized adverse effects were pain at the injection site (28.3%) followed by tenderness at the injection site (12.1%). In general, fever and body ache or malaise or weakness were the most commonly reported systemic adverse effects (33.5% and 29.2%, respectively) (Table 3). Fever, chills, headache and body ache, malaise, or fatigue were significantly associated with AZ compared to PF. Only allergic reaction, defined as a condition that developed within 24 hours of vaccine administration and needed medical attention/admission [16], was found to be associated with PF compared to AZ with 4/162 (2.4%) and 1/286 (0.2%), which developed an allergic reaction, respectively, p value = 0.006 (Table 3).

**Table 3:** Factors associated with reporting at least one adverse effect post-COVID-19 vaccination across various comorbidities

Clinical characteristics	Total sample, N = 753 (%)	Reported adverse effects post-COVID-19 vaccination		P value
		No, n = 378 (50.2%)	Yes, n = 375 (49.8%)	
Diabetes				0.310
No	496 (65.9)	215 (43.3)	281 (56.7)	
Yes	257 (34.1)	163 (63.4)	94 (42.6)	
Hypertension				0.141
No	454 (60.3)	204 (44.9)	250 (55.1)	
Yes	299 (39.7)	174 (58.2)	125 (41.8)	
Post-cancer				0.819
No	736 (97.7)	369 (50.1)	367 (49.9)	
Yes	17 (2.3)	9 (52.9)	8 (47.1)	
Hemoglobinopathies				0.349
No	735 (97.6)	367 (49.9)	368 (50.1)	
Yes	18 (2.4)	11 (61.1)	7 (38.9)	
Neurological disorder				0.806
No	738 (98.0)	370 (50.1)	368 (49.9)	
Yes	15 (2.0)	8 (53.3)	7 (46.7)	
Smoking				0.583
No	687 (91.2)	347 (50.5)	340 (49.5)	
Yes	66 (8.8)	31 (47.0)	35 (53.0)	
Immunosuppression				0.712
No	746 (99.1)	374 (50.1)	372 (49.9)	
Yes	7 (0.9)	4 (57.1)	3 (42.9)	
History of allergy				0.031
No	649 (86.2)	336 (51.8)	313 (48.2)	
Yes	104 (13.8)	42 (40.4)	62 (59.6)	
Asthma				<0.001
No	627 (83.3)	337 (53.7)	290 (46.3)	
Yes	126 (16.7)	41 (32.5)	85 (67.5)	
Alcohol consumption				0.296
No	702 (93.2)	356 (50.7)	346 (49.3)	
Yes	51 (6.8)	22 (43.1)	29 (56.9)	

**Figure 1:**
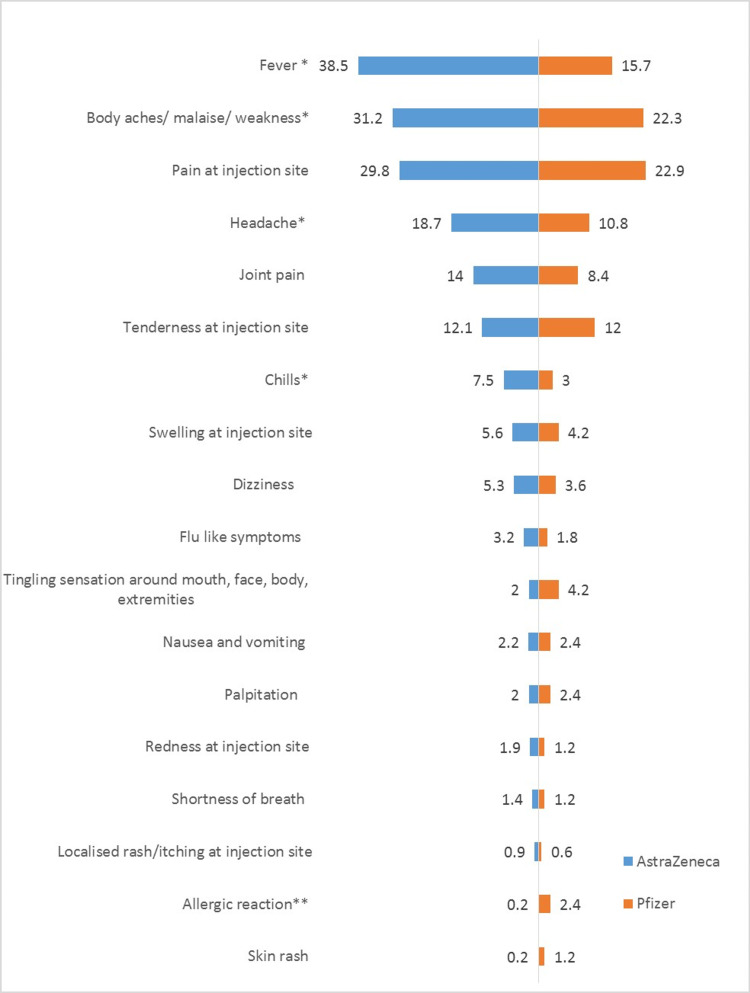
Reported adverse effects post COVID-19 vaccination (AstraZeneca vs Pfizer) * Adverse effects that are common with AstraZeneca vaccine. ** Adverse effects that are common with Pfizer vaccine.

An average of 85% of participants reported a recovery period of less than a week as a result of adverse effects. There was no significant difference in duration of adverse effects and type of vaccine except for headache and tenderness at the injection site. Results showed a longer duration of headache (of ≥7 days) in participants who received PF vs AZ (27.8% vs 5.5%, respectively, p value = 0.007) and longer duration of tenderness at the site of injection in the AZ group vs with PF (87.3% v 24.3%, p value = 0.029) (Figure 1).

Factors associated with reporting at least one side adverse effect post-COVID-19 vaccination

The proportion of participants with at least one side effect post-COVID-19 vaccination was significantly higher in younger versus older individuals, females versus males, individuals with more versus less than secondary education and employed versus non-employed individuals (p value < 0.001, 0.01, <0.001, and <0.001, respectively) (Table 1). Nationality (Omani vs non-Omani) and living status were not significant correlates for reporting at least one side effect (Table 1).

Additionally, the proportion of participants with at least one reported side effect post-vaccination was significantly more in individuals with a history of allergy and asthma (p = 0.03 and p < 0.001, respectively) (Table 3).

Perception of participants toward COVID-19 vaccines

When participants were asked about their perceptions of the safety of COVID-19 vaccines, the majority perceived the vaccines as safe (82.5%), and they would recommend it to others (94.4%). Results showed no significant difference between these perceptions and the type of vaccines.

## Discussion

Based on the epidemiological analysis of COVID-19 cases reported in Oman, the national COVID-19 vaccine committee recommends carrying out a nationwide campaign using two vaccine doses to protect the most vulnerable population [17]. The vaccine administration strategy was done in phases depending on the severity of disease, exposure risk, population susceptibility, and vaccine availability [18]. This is a rare study reporting data on the side effect of a COVID-19 vaccine in Oman to the best of our knowledge. It was conducted in the first stage of the campaign where the target group for vaccination was limited to age 60 and above and for a patient with asthma, chronic kidney disease, chronic lung disease, and healthcare workers. For that, participants aged ≥ 60 years constituted almost 60% of the participants of the study. However, the people aged 60 and above reported fewer adverse effects than the younger patient (p value < 0.001). A study from the United Kingdom published in *The Lancet* reported similar results; 46.9% (AZ) and 20.7% (PF) of people aged 55 years or younger reported at least one systemic effect after receiving their first dose, compared with 30.7% (AZ) and 10.6% (PF) of those older than 55 [16,19]. Younger adults may report more adverse effects, probably due to their robust immune systems, than older adults [16].

The proportion of females who reported at least one side effect was significantly more than males. Likewise, a study conducted by the Centers for Disease Control and Prevention reported 79% of adverse effects in women [19,20]. In general, women exhibit a greater vaccine-induced immunity, but they also experience more frequent and more severe adverse events [21-23].

Additionally, higher educational level and current employment status (including healthcare workers) showed significant association in reporting at least one side effect. This could be explained by the fact that education positively impacts persons’ behavior toward accepting vaccination and thus increases the alert and awareness to monitor any adverse effects/symptoms [24]. Similarly, employment may influence peer discussion on vaccination experiences and thus stimulate awareness of adverse effects, especially when early return to work is mandated [16].

The study also found a significant association between the reported adverse effects and the history of allergy and asthma. However, CDC recommends that people get vaccinated even if they have a history of severe allergic reactions not related to the administration of vaccines [25]. Moreover, more than 90% of individuals reported that adverse effects cleared within 48 hours after COVID-19 vaccinations [16].

During the study period, the vaccination of individuals previously infected with COVID-19 was deferred. However, 5% of study participants gave a history of the previous infection before vaccination. The effect of vaccination on the immunogenicity of individuals who encountered laboratory-confirmed COVID-19 is worth researching [16].

Generally, AZ was associated with more reported adverse effects than PF. This finding was reported in several studies, possibly due to AZ's mode of action as a live-attenuated vaccine and PF as an mRNA-engineered vaccine. Systemic adverse effects (diarrhea, fatigue, headache, chills, and nausea) affected fewer than one in four people. Still, they were more common with AZ, with at least one symptom reported by 33.7% after the first dose, compared to 13.5% and 22% after the first and second respective PF doses [16]. In comparing the adverse effects reported post-PF and AZ, a systematic side effect was more common with AZ, whereas allergic reaction was the most common post-PF. The possibility of reporting severe allergic reaction post-PF was reported in similar reports [26]. Existing evidence on dose-related adverse effects was more common after the second shot of the PF. At the same time, AZ recipients reported them more frequently after the first dose [27]. Nonetheless, most participants recovered in less than seven days despite a longer duration of headache post-PF [16].

Notably, no fatalities or serious adverse events were reported among the study participants. This finding may support the findings from the existing literature on the mild to moderate adverse effects of AZ and PF [26].

Irrespective of the type of vaccine, participants from this study perceived both the vaccines as safe and effective. They would recommend it to others, indicating modest acceptability to the vaccines.

There are several limitations to this study. First, recall or interviewer bias cannot be excluded, especially with interview-based surveys. However, to overcome it, the interviewers were trained on the questionnaire protocols before data collection. Second, this study was conducted at the first stage of vaccination. A number of participants who received two doses were too small to draw decent conclusions on a potential relationship between dose spacing and adverse effects. Third, the time period post-vaccination was short, and vaccine efficacy on the rate of infection was not studied. Finally, it may not be fair to compare the two vaccines considering differences in the timing of doses and coverage to different virus strains.

## Conclusions

This study looked at the adverse effects of COVID-19 vaccines and factors associated with reporting at least one side effect. Most study participants received AZ compared to PF. Most reported adverse effects were mild to moderate and resolved within ≤ seven days. However, AZ was associated with more systemic adverse effects, whereas PF showed more allergic reactions. Females, younger individuals, highly educated, and employed individuals were more likely to report at least one side effect post-vaccination. PF vaccine was associated with the most reported adverse effects after the second dose, whereas AZ showed more adverse effects after the first dose. Most participants perceived the vaccines as safe and effective, and they would recommend them to others. There were no serious allergic reactions or fatalities in this study. Finally, results from this study can be utilized to reassure the public on vaccine safety and thus reduce vaccine hesitancy.
